# A Minority Class Balanced Approach Using the DCNN-LSTM Method to Detect Human Wrist Fracture

**DOI:** 10.3390/life13010133

**Published:** 2023-01-03

**Authors:** Tooba Rashid, Muhammad Sultan Zia, Najam-ur-Rehman, Talha Meraj, Hafiz Tayyab Rauf, Seifedine Kadry

**Affiliations:** 1 Department of Computer Science, The University of Lahore, Chenab Campus, Gujrat 50700, Pakistan; 70076455@student.uol.edu.pk; 2 Department of Computer Science, The University of Chenab, Gujrat 50700, Pakistan; sultan.zia@cs.uol.edu.pk; 3 Department of Human Resource Section, University of Gujrat, Gujrat 50700, Pakistan; najam.rehman@uog.edu.pk; 4 Department of Computer Science, COMSATS University Islamabad-Wah Campus, Wah Cantt 47040, Pakistan; talhameraj32@gmail.com; 5 Independent Researcher, Bradford BD8 0HS, UK; hafiztayyabrauf093@gmail.com; 6 Department of Applied Data Science, Noroff University College, 4612 Kristiansand, Norway; 7 Artificial Intelligence Research Center (AIRC), Ajman University, Ajman 346, United Arab Emirates; 8 Department of Electrical and Computer Engineering, Lebanese American University, Byblos P.O. Box 13-5053, Lebanon

**Keywords:** augmentation, convolution neural network, human wrist fracture, long short-term memory, lifesaving

## Abstract

The emergency department of hospitals receives a massive number of patients with wrist fracture. For the clinical diagnosis of a suspected fracture, X-ray imaging is the major screening tool. A wrist fracture is a significant global health concern for children, adolescents, and the elderly. A missed diagnosis of wrist fracture on medical imaging can have significant consequences for patients, resulting in delayed treatment and poor functional recovery. Therefore, an intelligent method is needed in the medical department to precisely diagnose wrist fracture via an automated diagnosing tool by considering it a second option for doctors. In this research, a fused model of the deep learning method, a convolutional neural network (CNN), and long short-term memory (LSTM) is proposed to detect wrist fractures from X-ray images. It gives a second option to doctors to diagnose wrist facture using the computer vision method to lessen the number of missed fractures. The dataset acquired from Mendeley comprises 192 wrist X-ray images. In this framework, image pre-processing is applied, then the data augmentation approach is used to solve the class imbalance problem by generating rotated oversamples of images for minority classes during the training process, and pre-processed images and augmented normalized images are fed into a 28-layer dilated CNN (DCNN) to extract deep valuable features. Deep features are then fed to the proposed LSTM network to distinguish wrist fractures from normal ones. The experimental results of the DCNN-LSTM with and without augmentation is compared with other deep learning models. The proposed work is also compared to existing algorithms in terms of accuracy, sensitivity, specificity, precision, the F1-score, and kappa. The results show that the DCNN-LSTM fusion achieves higher accuracy and has high potential for medical applications to use as a second option.

## 1. Introduction

A bone fracture happens when a high force is applied against a bone. Fractures of a bone can be caused by trauma, osteoporosis, and overuse. According to a recent study by the World Health Organization (WHO), a fracture affects a significant number of people, and the consequences of a neglected fracture can lead to severe injury or even death. A wrist fracture is a quite common injury, and positive cases are increasing on a daily basis [1]. Wrist fracture can be the result of an accident, such as slipping over with an outstretched palm. Often, extensive injuries occur from intense physical trauma, such as automobile accidents or falls from a roof. In osteoporosis, weak bone appears to crack more swiftly. Countless incidences need healthcare professionals to examine fractures. Both human and environmental factors, such as inexperienced physicians, physical fatigue, distractions, poor observation circumstances, and time constraints, promote radiographic analysis errors.

The advent of radiographic technology has significantly improved the early diagnosis of fractures. Some medical imaging technologies, such as X-ray, magnetic resonance imaging (MRI), computed tomography (CT), and ultrasound, are convenient to capture fractures. However, with relatively low cost and accessibility, X-rays are most generally implemented in bone fracture diagnosis. However, in comparison to the enormous incidence of fractural cases, the lack of skilled radiologists is huge. As a result, many radiologists are fatigued by the massive amount of medical imaging [2]. To overcome this concern, computer-aided diagnostic (CAD) technology has been used to assist physicians to analyze medical images. Recent studies have shown that incorporating machine learning and deep learning methods into CAD systems enhances the performance of healthcare professionals.

Machine learning is a sub-domain of artificial intelligence (AI) that uses various algorithms, such as a support vector machine (SVM) [3], decision tree (DT), K-nearest neighbour (KNN) [4], neural network (NN), and random forest (RF) [5], for automatic fracture diagnosis, classification, and prediction. However, when the number of features is significantly beyond the number of observations, these algorithms produce inadequate outcomes, which cannot fulfil the potential medical need. Nowadays, deep learning has emerged as the most powerful AI technology in the medical field [6].

Deep learning enables raw data to be seamlessly transferred into simulators and analyzed with multiple feature extraction and weighting levels [7]. Deep learning is widely used for its immense potential in intricate feature extraction and predictions in a variety of medical fields, including pathology [8], pharmacology, radiology, ophthalmology, and even feature detection, including both ulnar and radial fractures [9,10], pulmonary tuberculosis classification [11], cancer diagnosis [12], rib fracture [13], hip fracture [14] hand and wrist fracture [15], ankle fracture [16], and thoracolumbar fracture [17]. Convolutional neural networks (CNNs) tend to be a more efficient strategic method for feature detection and have rapidly acquired significance in the computer vision area in the past years. To modify clinical challenges, CNNs “train” distinguishing patterns using models, such as Inception V3 [18], ResNet [19], U-Net [20], Xception [21], and DenseNet [22]. Due to limited annotated data availability, data augmentation and other data generative methods can be used to increase the size of the dataset. The interpretation of a deep learning algorithm is still a challenge since its accuracy is highly dependent on the features of the learned data, including diagnostic precision and fracture extent. In general, specific input data and modification are frequently needed for pre-existing deep learning techniques before proposing any clinical development.

To automatically diagnose wrist fracture from X-ray images, this research attempts to provide a deep learning method that adopts the CNN and LSTM networks together to enhance the patient’s experience by enhancing performance, minimizing misdiagnosis, and reducing delayed treatment. Clinicians depend on X-ray images to detect where the fractures have occurred. The earlier approach could only identify fractures in a specific bone area, such as the distal radius [10]. However, various forms of bone fractures can be seen in an X-ray image.

The contributions of this study are mentioned next.

(a) To alleviate clinicians’ cognitive load, the two-method model adopted in this research detects significant fracture areas in an X-ray image.
(b) A fusion method, DCNN-LSTM, is proposed that can automatically diagnose wrist fractures to facilitate the radiologist.
(c) The first model, the DCNN, is designed to enhance the CNN’s receptive field by using a dilation factor in the convolution operation, which lessens the complexity of the CNN training phase.
(d) The fused model DCNN-LSTM enhances accuracy with augmented data to recognize anomalies using wrist X-ray images.
(e) The entire research work is structured as follows: Section 2 presents related works on fractures. Section 3 provides an overview of the proposed methodology, comprising dataset acquisition and analysis. Section 4 includes a discussion of the results and an evaluation of the proposed methodology. Section 5 concludes the research work.

## 2. Related Works

Machine learning (ML) and deep learning (DL) enable AI systems to take a step ahead as they allow cognitive learning to take place within a framework dependent on prior studies or data analysis. As it progresses, the model executes challenging decision methods and evaluates past activities. Researchers have designed deep learning approaches to detect fractures, depending on clinical radiographs, CT scans, and X-rays of bone. Various bone fracture detection and classification methods have been proposed traditionally. However, they are statistical methods, they cannot detect the fracture location in the bone, and they need a number of pre-processing stages. This study discusses recently developed methodologies that use different machine learning and deep learning approaches to detect fractures.

**Figure 1:**
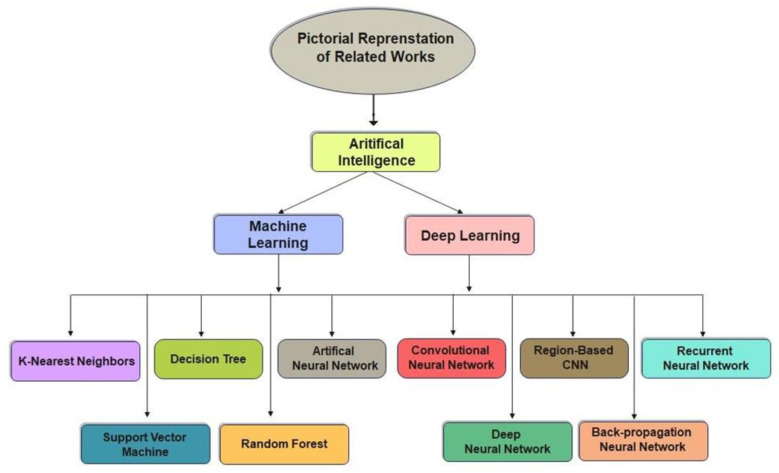
Pictorial representation of related works.

Various imaging approaches are being developed to identify lower leg bone (tibia) fracture variants. Myint et al. [23] used the machine learning algorithms K-nearest neighbour (KNN) and decision tree (DT) to detect tibia fracture from X-ray images. The chest CT images of 1707 patients were used by Yao et al. [24] to classify rib fractures using a three-step algorithm and achieved an accuracy of 0.869 on 4496 CT images. The most popular object recognition deep learning model YOLOv3 was used by Choi et al. [25] to diagnose skull fractures in X-ray images and achieved an accuracy of 91.7% on a limited dataset. Tomita et al. [26] identified incidental OVFs in chest, abdomen, and pelvic CT scans. The OVF detection system uses a deep convolutional neural network (CNN) to accumulate radiological features out of each CT slice, and the resulting features are then evaluated using the long short-term memory (LSTM) network to determine the final decision for a complete CT scan report. This approach achieved an accuracy of 89.2% and has the potential to minimize the time and cognitive load on radiologists for OVF monitoring, along with the risk of adverse outcomes. Kim et al. [27] used transfer learning from deep CNNs to diagnosis wrist fractures from X-ray images in four stages. The findings showed that the model performs efficiently on a small dataset. The findings of the system using deep learning techniques and level-set methods by Kim et al. [28] achieved efficient results on 160 lumbar X-ray images to identify and segment each lumbar vertebra. Guan et al. [29] used a novel deep learning model fast R-CNN to diagnose arm bone fractures on X-ray images. The algorithm fast R-CNN was trained on the MURA dataset comprising 4000 X-ray images with low quality and achieved a state-of-the-art average precision of 62.04% for diagnosing arm fractures, which is much faster than existing state-of-the-art deep learning methods. CNN is used in combination with other modules to recognize, localize, and categorize objects in images. Thian et al. [30] applied the Inception-ResNet Faster R-CNN model on 7356 wrist images. Even before conducting classification, they trained their algorithm on the wrist imaging dataset. For adequate and comprehensive classification and contextual localization of radius and ulna fractures in wrist radiographs, an object detection deep learning approach was optimal and achieved an accuracy of 91.8%. Guan et al. [31] introduced a novel deep learning technique termed “dilated convolutional feature pyramid network” (DCFPN) to identify thigh fracture. The algorithms were trained using 3484 X-ray images from Linyi People’s Hospital, and the learning algorithm was tested using 358 images. The DCFPN findings outperformed traditional algorithms of deep learning. The overall related work algorithms regarding fracture detection in different body organs are summarized in a visual illustration in Figure 1.

Ebism et al. [32] detected the radius in both posteroanterior PA and lateral wrist images using a random forest (RF) classifier. Guo et al. [33] collected CT images of orbital blowout fractures from the Shanghai Ninth People’s Hospital and used the Inception-V3 convolutional neural network (CNN) framework with the XGBoost model to classify the orbital blowout fractures. Zeelan et al. [34] detected bone fracture from X-ray images using the machine learning models probabilistic neural network (PNN), backpropagation neural network (BPNN), and support vector machine (SVM) and classified the input images into the classes skull, head, chest, hand, and spine. A deep convolutional neural network (DCNN) model developed by Cheng et al. [35] not only detected hip fractures on plain frontal pelvic radiographs (PXRs) with a good accuracy rate, but it was also good at localizing fracture areas. In the human body, bone fractures are common. Rathor et al. [36] used a deep neural network (DNN) to detect fractured bone from X-ray images and achieved a 92.44% accuracy, but the model still involves affirmation on a huge dataset. Ebsim et al. [37] trained a convolutional neural network (CNN) to detect wrist fractures in posterioanterior (PA) and lateral radiographs. Adigun et al. [38] combined two machine learning models, K-nearest neighbour (KNN) and support vector machine (SVM), to enhance each other’s findings. The KNN-SVM attained better classification accuracy than earlier work. Myint et al. [39] trained support vector machine (SVM) on 40 X-ray images to classify two types of fractures (non-fracture and fracture or transverse fracture). According to the paper’s conclusions, good outcomes were not achieved. Mondol et al. [40] built a model using VGG-19 and ResNet to detect four types of fractures: elbow, wrist, finger, and humerus on the MURA dataset. Lindsey et al. [41] developed a deep convolutional neural network (DCNN) model to detect and locate fractures in 135,845 PA or LAT wrist, foot, elbow, shoulder, knee, ankle, pelvis, hip, humerus, and shoulder radiographs. Chittajallu et al. [42] used a CNN model that was trained on 200 images of human hands, ribs, legs, and the neck to detect fracture, and the findings achieved adequate predictions. Vasilakakis et al. [43] proposed a novel approach using wavelet fuzzy phrases (WFP) for feature extraction and classification. The classification performance of the model achieved better results, potentially minimized diagnostic errors, and improved the radiologists’ performance. Dimililer et al. [44] designed an intelligent classification system using a backpropagation neural network (BPNN) that was capable of detecting and classifying bone fractures from 100 X-ray images. Hržić et al.’s transfer learning model YOLOv4 was used to detect wrist fractures from X-ray images. The functional testing on three aspects demonstrated that the YOLOv4-based model significantly outperforms the state-of-the-art technique based on the U-Net framework and achieves better accuracy [45].

The summary of recently applied studies is shown in Table 1. All studies used different datasets on different organs of the human body to propose an efficient method for fracture detection. However, the studies did not mainly consider the class imbalance problem and annotated data availability to fulfil the requirement of deep learning input data. In addition, many of the studies did not consider multi-model usage to include multi-feature usage for precise detection of body fractures. Therefore, the proposed study firstly solves the class imbalance problem and secondly applies the multi-feature extraction approach to precisely classify wrist fractures.

## 3. Proposed Methodology

**Figure 2:**
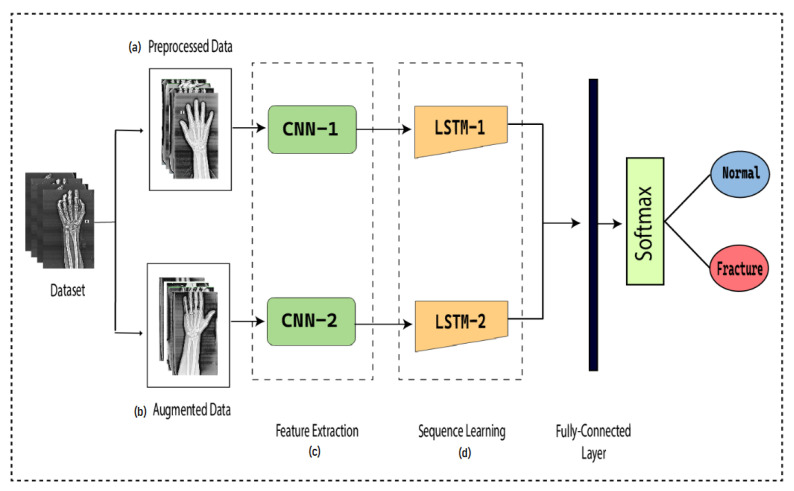
DCNN-LSTM main parts. (**a**) X-ray images are resized and enhanced in the pre-processing stage. (**b**) The diversity of the dataset increases in the data augmentation stage. (**c**) The dilated-CNN model for feature extraction from wrist X-ray images. (**d**) Extracted features are classified into normal and fractured images through the LSTM network.

To detect the presence of fractures from the wrist images of humans, we designed a combination of a dilated convolutional neural network (DCNN) and an LSTM network. The proposed network, DCNN-LSTM, has two methods, as illustrated in Figure 2. X-ray images of patients were acquired from the Mendeley data repository. Firstly, the images were passed through the pre-processing stage. Image resizing and enhancement were performed in the pre-processing stage. Secondly, the data augmentation approach was used to enlarge the dataset. Next, pre-processed images and augmented normalized data were fed into a 28-layer dilated CNN to extract valuable features and eliminate features that were not beneficial. Finally, the LSTM network was used to accurately classify the normal and fractured images.

### 3.1. Data Normalization and Pre-Processing

Normalization of data has always been an essential aspect of the pre-processing method, focused on eliminating data redundancy and inconsistency from the database in order to regulate the complexity of the network and provide accurate findings. The images captured in real time are of various sizes and destroyed for numerous factors. An image must be resized to accommodate the size of the model for which it is built. The X-ray images used in this study were in JPG format with different sizes. All images were resized to ones with a resolution of 512 × 256 × 3 pixels using the bi-cubic interpolation method. In the bi-cubic method, 4 × 4 neighbouring pixels were used to evaluate the weighted average. The pixels that were nearest in both vertical and horizontal directions were weighted in computations. Assuming that $\left(x,\;y\right)$ are the coordinates of the point from which we aimed to explore the new pixel, the value was determined using the following equation:

(1) $$v\left(x,y\right)=\overset{3}{\underset{i=0}{\sum }}\overset{3}{\underset{j=0}{\sum }}a_{ij\;\;\;\;\;x^{i}\;y^{j\;}\;}$$

To increase the brightness of the X-ray images, the image contrast enhancement method is effective to apply. Image enhancement was accomplished by modifying the image’s histogram attributes, altering the image’s histogram, enhancing the various grey levels, and increasing the image’s contrast. To reduce data loss and distortion, we used the adaptive histogram equalization method in this study. It enhances the contrast of the image and edges to detect the area of interest.

### 3.2. Data Augmentation

Data augmentation, which estimates the data probability space by modifying input images, such as rotation, random crop, scaling, and noise disturbance, is an efficient and effective strategy to reduce the “overfitting” of the deep convolutional neural network due to inadequate training images. The model’s performance can be enhanced in general by increasing the volume, quality, and variety of data in the dataset. The method used to augment images in this study was the affine transformation method. Affine transformation enhances the alteration of an image’s geometric structure by maintaining line parallelism but not dimensions and angles. It uses a linear collection of translation, rotation, scaling, and/or shearing operations to process data into new data. In this study, we applied a rotation operation of affine transformation on the images to increase the size of the dataset. Rotation is most often optimized to enhance an image’s visual effect, but it can also be applied as a modifier in systems that use directional operators. To rotate the image around a centre or an axis, we simply provide the angle of rotation. The angle of rotation is denoted by  θ , which specifies how many degrees the image is rotating.

(2) $$R=\left[\begin{array}{ccc} \cos\left(\theta \right) & \sin\left(\theta \right) & 0\\ -\sin\left(\theta \right) & \cos\left(\theta \right) & 0\\ 0 & 0 & 1 \end{array}\right]$$

The original image is mapped to a new image by rotation at an angle of 0°, 90°, 180°, 270°, and 360°. The rotation operation increases the diversity of the dataset and thus enables the deep learning model to identify the data intrinsic features quite comprehensively. Training based on augmented data can potentially enhance the performance of a deep CNN as compared to training without data augmentation.

### 3.3. Proposed Dilated CNN

**Figure 3:**
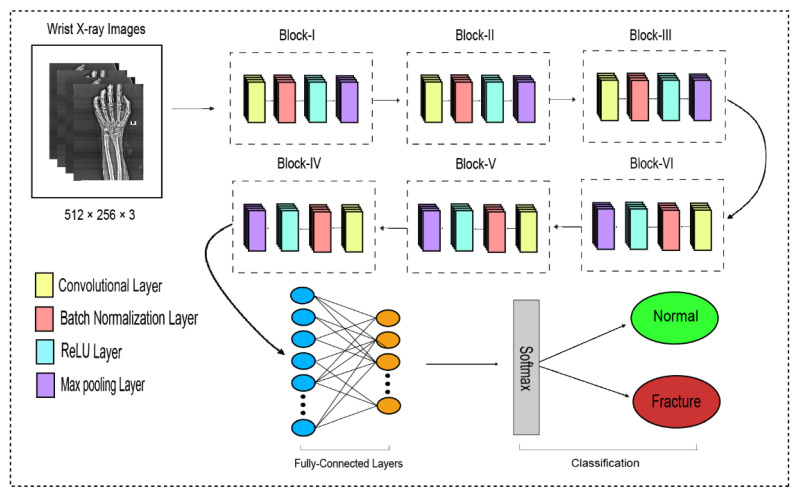
The dilated convolutional neural network for wrist fracture detection.

The traditional CNN’s primary function is to execute the convolution operation on the specific region of an image. The input layer, convolutional layer, batch normalization, ReLU, pooling layer, fully connected layer, and output layer constitute the CNN, as shown in Figure 3. When the number of feature channels increases, the number of parameters of the convolution kernel escalates as well, resulting in an increase in simulation. The standard convolutional neural network (CNN) was substituted in this study by the dilated convolution neural network (D-CNN). The D-CNN uses larger two-dimensional filters. Traditional CNNs often use smaller convolution filters (typically 2 × 2 or 3 × 3). The D-CNN uses dilated convolution filters, which are enlarged filters. A dilated convolution with a dilatation rate r comprises r-1 zeros between consecutive filter values, enlarging the size of a k × k filter to $\left[k+\left(k-1\right)\left(r-1\right)]\;*\;[k+\left(k-1\right)\left(r-1\right)\right]$ [46]. The dilated convolution filters expand the CNN’s receptive field without integrating any additional parameters. Thus, the D-CNN uses slightly fewer layers than the CNN to attain the same receptive size of the field, preventing the overfitting problem caused by a deep CNN.

The proposed dilated convolution neural network (D-CNN) was based on a 28-layer architecture and had six blocks. The size of the input images was set to 512 × 256. The pre-processed images were fed into D-CNN1, and augmented images were fed into D-CNN2, where (convolution, batch normalization, rectified linear unit, and max pooling) were performed with different parameters, as shown in Table 2. The input layer is the initial layer and feeds images to the system. The next layer is the convolution layer, which executes the convolution process on the obtained images by simply shifting a window across it with a stride. For our initial convolution layer, we constructed a 5 × 5 window and used kernels to convolve the images; the number of filters was set to 16, and the amount of filtering increased as further convolutional layers were included. The receptive field size was enlarged by a dilation factor in the C2, C3, C4, C5, and C6 layers. Batch normalization was applied to normalize the previous layers’ outcome and to avoid overfitting. Batch normalization facilitates each layer of the system to perform learning autonomously. After the convolution process is completed, the subsequent feature maps are used as input to the batch normalization layer, which is preceded by the rectified linear unit, which uses the function $f\left(x\right)=\max\left(0,x\right)$ . The basic concept to use this function *f*(*x*) is to set the values of neurons that are above a certain point to zero. This minimizes data redundancy and preserves essential features. A feature layer’s size can be lessened by using max pooling on each feature map with stride 2 × 2. All neurons in the fully connected layer are considered and associated with the neurons in the preceding max-pooling layer. The number of output classes is determined by the output from the fully connected layer, which is preceded by the softmax layer.

Input Layer:

Layers follow the tensor to modify and then rebuild the tensor in this layer. The input layer is the entire DCNN’s input. It widely represents an image’s pixel matrix in the deep learning of image processing, and the image’s pixel values are kept in this layer. An image input layer feeds images to the system. A DCNN uses tensors of the shape image height, image width, and colour channels as input. In our proposed system, this was 512 × 256 × 3, where the height of an image is 512, the width of an image is 256, and the number of colour channels is 3.

Convolution Layer:

The convolutional layer’s primary function is to identify local conjunctions of features from the preceding layer and transfer their structure to a feature map. The input image is processed by a filter in this layer. By changing the dilated rate, the dilated convolution enlarges the receptive field of the convolution kernel. As a consequence, dilated convolution achieves multi-scale information by enlarging the receptive field of the convolution kernel. Whenever the dilation rate is 1, the receptive size of dilated convolution remains equivalent to that of conventional convolution. In our study, we applied six convolution layers with a dilation rate r that increased the size of the filter k × k to extract the features from the input images. The formula stated next is applied to compute the receptive field size of a dilated convolution [47,48]:

(3) $$S=F_{c\;}+\left(F_{c}-1\right)\left(R-1\right)$$

S denotes the receptive field size, and R denotes the size of the dilation factor. When the dilation value is far more than 1, dilated convolution can acquire a relatively large receptive field size and accumulate more diverse visual information compared to conventional convolution. When numerous dilated convolutional layers are deployed in the framework, the receptive field size of the convolution kernel is determined layer by layer according to the conceptual framework, with the assumption that the dilation factor is specified appropriately.

Our first layer was a convolution layer comprising 16 feature maps and a 5 × 5 kernel size. Secondly, the convolution layer comprised 32 feature maps with a 5 × 5 kernel size with a dilation rate r = 2. Third was a convolution layer comprising 64 feature maps with a 5 × 5 kernel size with a dilation rate r = 4. Fourth was a convolution layer of 128 feature map with a 5 × 5 kernel size with a dilation rate r = 8. Fifth was a Convolution layer of 128 feature maps with a 5 × 5 kernel size with a dilation rate r = 16. Sixth was a convolution layer of 256 feature maps with a 5 × 5 kernel size with a dilation rate r = 32.

Batch Normalization Layer:

When the convolution process is completed, the cumulative feature maps are obtained and used as input to the batch normalization layer. Batch normalization facilitates each layer of the system to perform learning autonomously. The batch normalization method normalizes the input value $x_{i}\;$ by first estimating μB and $\;\sigma_{B}^{2}$ , along with a small batch size to boost CNN training, while significantly reducing the network initialization accuracy. The normalized activations are then calculated using the following equation:

(4) $$\hat{x_{i}}=\frac{\;x_{i}-\;\mu B}{\sqrt{\sigma_{B}^{2}+e}}$$

The batch normalization layer’s normalized outcome is maintained in $\;\hat{x_{i}}$ . The outcome of the batch normalization layer is then used by the ReLU activation function.

Rectified Linear Unit (ReLU):

The ReLU function was used as the activation function in each convolutional layer to increase non-linearity in the yield, and it is represented as:

(5) $$f\left(x\right)=\max\left(0,x\right)$$

It is a non-linear operation that transmits zero outcomes when handed a negative input and one outcome when handed a positive input. The ReLU has no finite possibilities for positive input values, and the gradients are either zeros or ones. This permits the ReLU to evaluate efficiently and yield relatively high accuracy.

Pooling Layer:

By conducting dimension reduction, the pooling layer strives to decrease the number of parameters in the convolutional yield. In our study, we applied max pooling with a 2 × 2 kernel size and with a stride of 2. The layer in max pooling typically works with the most significant feature in the feature map obtained from the convolutional layer.

The pooling layer accepts the input size of $W_{1}\times H_{1}\times D_{1}$ from the convolutional layer, where $W_{1}$ is the width, $H_{1}$ is the height, and $D_{1}$ is the depth. The pooling layer’s window size in each block remains the same at a 2 × 2 kernel size and a stride of 2. The pooling layer needs two hyperparameters: the spatial extent ( F ) and the stride ( S ). The MAX operation resizes each slice of the input spatially.

The output size of the pooling layer is $W_{2}\times H_{2}\times D_{2}$ , where:

(6) $$W_{2}=(W_{1}-F)/S+1$$

(7) $$H_{2}=\frac{\left(H_{1}-F\right)}{S+1-F\;}\;\;$$

(8) $$D_{2\;}=D_{1}$$

Fully Connected Layer:

The fully connected layer connects each neuron from the preceding layer to each neuron from the succeeding layer. The output acquired from the preceding convolutional layer and pooling layer is generally flattened, which is transformed into a 1−D array and associated with two fully connected layers. We attached the outcome extracted by the max-pooling layer to a fully connected network comprising two layers to complete the DCNN-BiLSTM model.

Softmax Layer:

The softmax layer is used to categorize the feature map outcome of fully connected layers, and the outcome is a vector indicating probability classification, with values varying from 0 to 1. The softmax activation function is mathematically represented as:

(9) $$\sigma \left(x_{i}\right)=\frac{e^{xi}}{\sum_{i=0}^{n}e^{xi}}$$

where $x_{i}$ represents the *i*-th element in array  x and i represents the total number of elements in array  i .

### 3.4. Feature Extraction

The pre-trained DCNN model’s output structure consists of a fully connected layer and a softmax classifier. Convolving the fully connected layers of the proposed DCNN model yields the feature vector. The inclusion of the fully connected layer at the end of the DCNN has a number of significant advantages. In the concluding stage of the architecture, the feature map generated from the DCNN is transferred through transfer learning to the LSTM layer to retrieve time data. Deep features obtained from the pre-trained DCNN are transferred into a new process without any need to train the new process with a tremendous number of labelled data values, which is particularly accurate and convenient for image classification.

### 3.5. LSTM

An LSTM network is a recurrent neural network variant that uses memory blocks to operate more effectively and learn more efficiently than a traditional RNN. LSTM networks provide feasible alternatives to RNNs’ vanishing and exploding gradient concerns. An network is composed of a sequence of memory blocks termed “cells,” each of which contains three gates: input, output, and forget. Therefore, the LSTM network can remember the prior data and relate it to the current data. It also handles complex tasks for which prior RNNs were inadequate to discover a solution [49]. These gates enable information to flow through selectively. In other words, using the three gates (input, output, and forget), the LSTM can regulate which information is maintained, which information is rejected, and which information is delivered. The LSTM represents unidirectional input data; therefore, it cannot preserve structural localization and is more vulnerable to overfitting. It has a hidden layer h in the forward direction that processes the input from left to right by acquiring the left context of the current input. At each input sequence, an input vector is given into the LSTM, and the output is generated based on:

(10) $$h_{t}=f\left(\;h_{\left(t-1\right)},\;x_{t}\right)$$

where $\;x_{t}$ represents the input state, $h_{t}$ represents the present state, and $h_{\left(t-1\right)}$ represents the prior state.

### 3.6. Bidirectional LSTM

Although the LSTM is unidirectional, it only acquires a small number of the context, which significantly reduces the classification performance. To improve the classification performance of the long-term dependencies of time series data without affecting latency is to handle the information bidirectionally [50]. A BiLSTM uses various layers to handle contextual information in both directions, forward and backward. It has a hidden layer with the forward sequence $\vec{h}\;$ and the backward sequence $\overset{\leftarrow }{h}$ for the left and right contexts, respectively [51]. It must be ensured that each subsequent layer is taken from both the forward and backward layers. In the proposed network, the output of the CNN was fed into the BiLSTM layer to accurately the normal and fractured images. Let’s look more deeply at BiLSTM layers and thus at how they operate independently.

Sequence Input Layer

The BiLSTM network begins with a sequence input layer, and it is the primary feature of a BiLSTM network. A sequence input layer is responsible for transmitting sequential data to a network. The input size of a sequence input layer is 2.

BiLSTM Layer

DCNN findings are transferred to the BiLSTM layer for further feature extraction. Figure 3 illustrates two LSTM layers. The BiLSTM is structured with a forward and a backward LSTM layer. The forward LSTM may acquire preceding information from the input sequence, while the backward LSTM can acquire forthcoming information from the input sequence, and the results from both hidden layers are then integrated.

(11) $$h_{t}=\;\vec{h}\;⊕\;\;\overset{\leftarrow }{h}$$

where ⊕ symbolizes the feature computation used to add the forward and backward output features. The first BiLSTM layer contains 2000 hidden units, whereas the subsequent BiLSTM layer contains 1000 hidden units. Although it can use both prior and subsequent knowledge, a BiLSTM is more suitable than the LSTM and RNN.

Dropout Layer

A dropout layer is integrated after both BiLSTM layers to avoid the overfitting problem; the dropout rate is 0.5, signifying that 50% of the information is discarded. The decrease of 1000 hidden units after the first layer of the BiLSTM and the decrease of 1000 hidden units after the second layer of the BiLSTM are done to avoid overfitting.

Fully connected layer

We attached the outcome extracted by the BiLSTM layer to a fully connected network comprising two layers to complete the DCNN-BiLSTM model.

Softmax Layer

**Figure 4:**
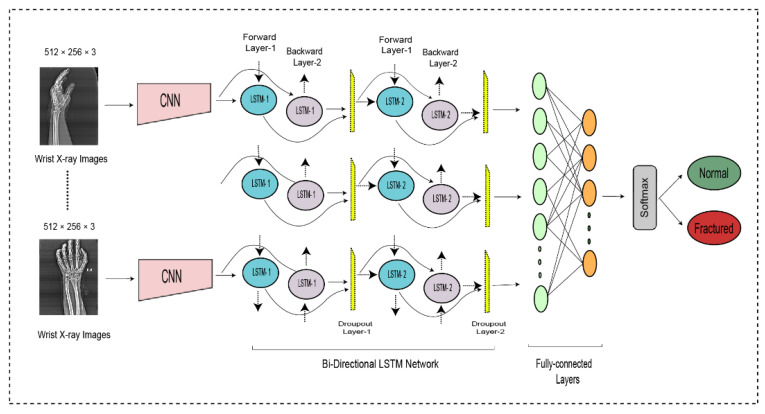
The bi-directional LSTM network for wrist fracture detection.

The entire DCNN-BiLSTM architecture is shown in Figure 4. At the end of the architecture, the softmax activation function was used to classify the output from the preceding layer. The softmax layer predicted the class of the images as fractured or non-fractured. Table 3 represents the parameter setting for proposed bidirectional LSTM.

## 4. Results and Discussion

The proposed framework used a dataset with its original images at first in experiment 1 and then applied data augmentation to solve the class imbalance and data overfitting problem for deep learning in experiment 2. Both experiments used a different number of images but the same methods of classification. In each experiment, the DCNN method was applied to extract deep features and then fed to the BiLSTM network for classification. In total, two experiments and four methods were applied, with two methods in each experiment.

### 4.1. Dataset Description

The wrist fracture X-ray images of patients used in our research were acquired from the Mendeley dataset, which is openly available. Mendeley collects X-ray images from the Al-huda Digital X-ray Laboratory at Nishtar Road in Multan, Pakistan. The dataset consists of 111 fractured and 82 normal wrist X-ray images. The images captured in real time are of various sizes and in JPG format. All images were resized to ones with a resolution of 512 × 256 pixels to use in the system, and the dataset before and after pre-processing and augmentation is shown in Table 4.

### 4.2. Performance Evaluation

In this work, after applying pre-processing, data augmentation, feature extraction and classification were performed on the dataset. The last stage was to determine the model’s performance. There are four potential outputs when using a classifier on any case. These outputs are:

- True-positive (TP) outputs include positive images (fractured) that are accurately classified as fractured.
- True-negative (TN) outputs include normal images that are accurately classified as non-fractured.
- False-positive (FP) outputs include normal images that are inaccurately classified as fractured.
- False-negative (FN) outputs include positive images (fractured) that are inaccurately classified as non-fractured (normal).

The main purposes of measuring a classification model’s prediction outcomes are (1) to evaluate the overall performance and (2) to improve the classifier’s predictive potential by modifying the input variables of the model. In the mentioned description, the performance of the presented approach was analyzed in terms of accuracy, precision, sensitivity, specificity, the F-score, and kappa. Sensitivity indicates the percentage of all positive cases detected and evaluates the classifier’s potential to detect positive cases, whereas recall is similar to sensitivity. Specificity reflects the percentage of all normal cases detected and indicates the classifier’s capacity to comprehend normal cases. Precision shows the percentage of positive classes that are divided into positive cases. The F1-score is a comprehensive evaluation metric, and a significant value shows that the classifier is much more accurate. The Cohen kappa metric improves assurance in the outcomes. The formulas are mentioned next.

(12) $$Accuracy=\frac{TP+TN}{TP+TN+FP+FN}$$

(13) $$Precision=\frac{TP}{TP+FP}$$

(14) $$Sensitivity=\frac{TP}{FP+TN}$$

(15) $$Specficity=\frac{TN}{FP+TN}$$

(16) $$F1-Score=\frac{2\times \left(TP\right)}{\left(2\times TP+FN+FP\right)}$$

(17) $$Kappa=\frac{2\times \left(TP\times TN-FN\times FP\right)}{\left(TP+FP\right)\times \left(FP+TN\right)+\left(TP+FN\right)\times \left(FN+TN\right)}$$

### 4.3. Experiment 1 (Non-Augmented Data)

To enhance the efficiency and results of the designed model, pre-processing was used in the first method in this study. As it is a major part of the classification model, the quality and significant knowledge gathered in the pre-processing process have a significant influence on the model’s training. The experimental results obtained after applying the dilated CNN to the pre-processed data achieved an 84.48% accuracy, 87.50% precision, 84.85% sensitivity, 84% specificity, 86.15% F1-score, and 68.52% Cohen kappa. If we look at the different scores of the results achieved, we can analyze the true-positive over the false-positive score, which is precision, which was more than the accuracy score. However, the sensitivity and specificity were nearer to the accuracy score, which are rates of true positives and true negatives, respectively. The F1-score was higher at 86.15% compared to accuracy, which is good as it covers the class-imbalance-addressing measure too.

To enhance the outcomes of the dilated CNN, we transferred the findings into the LSTM model and achieved an accuracy of 86.21%, which increased as compared to the DCNN method, although the purpose of the implemented LSTM is shown here as increased results by just feeding already trained features using the DCNN method. The sensitivity, F1-score, and Cohen kappa rates significantly improved in this method, whereas other measures remained the same. The results are shown in Table 5.

### 4.4. Experiment 2 (Augmented Data)

In experiment 2, to increase the diversity of the dataset and to resolve the concerns of overfitting, the data augmentation method was applied in this study. The rotation operation of affine transformation was applied on the X-ray images’ dataset. Both methods, dilated CNN and LSTM, were again applied to enhance the model’s accuracy by increasing the volume, quality, and variety of data in the dataset and thus enable the proposed model to identify the data’s intrinsic features quite comprehensively. The experimental results obtained after applying the dilated CNN on the augmented data achieved an accuracy of 86%, which is better than that of DCNN1 applied in experiment 1 on the non-augmented dataset. Similarly, other scores, such as sensitivity, F1-score, and Cohen kappa, also increased significantly. To improve the DCNN2 results, we transferred the observations into the BiLSTM. By using the BiLSTM, the model obtained an accuracy of 88.24%, which is better than that of the DCNN-LSTM1 methods applied in experiment 2. The other scores of the DCNN-LSTM 2 also improved and achieved up-to-the-mark results for wrist fracture detection. Training based on augmented data enhanced the computational performance of the model as compared to training without data augmentation.

The tabular description of experiment 2 results is shown in Table 6. The improvement of results showed that the model performance in terms of precise results improved by implementing a 2-step approach of classification by integrating CNN and LSTM methods.

## 5. Conclusions

Many countries are experiencing resource limitations as fracture cases are increasing on a regular basis. It is essential to detect every individual positive case. The increasing rates of accidental cases have increased the need for medical staff in the emergency department, which is badly needed in many lower-income countries. The existing staff is less in number, and due to a shortage of doctors, the overload could lead them to less accurately detect fractures. Therefore, there is a need to develop a computer-assisted method for detection of body organ fractures in order to facilitate the second-option diagnosis for doctors. This study proposed a CAD system based on a DCNN-LSTM network approach to detect wrist fractures from X-ray images. For the proposed research, a dataset of 111 fractured and 82 normal wrist X-ray images from Mendeley was used. Image data pre-processing operations were applied; for example, image enhancement was used to enhance the brightness of the X-ray images and to reduce the distortion. To prevent overfitting and to increase the diversity of the dataset, the data augmentation technique was used. We attempted to maintain the class balance by generating an equal number of images for both classes during the training process. Two experiments were conducted in this study to prove the improvement in results using data augmentation, in which a deep learning method named “dilated CNN” was used to enhance the receptive field of the CNN with a dilated factor to extract valuable features from X-ray images. Secondly, an LSTM network was used as a classifier for the detection of wrist fracture. Furthermore, the capabilities of the proposed DCNN-LSTM models with and without augmentation were compared with those of other deep learning models. The overall accuracy of the proposed model with augmentation was 88.24%, and it was almost more than that without augmentation (86.21%). As the incidence of fracture cases continues to increase, we believe that our model will be useful for medical evaluation. An automated detection system will improve detection and classification and will contribute to the solution of the incident load issue faced by medical experts.

However, the limitations of data could be a limitation for the proposed method, as with increasing data, the applied approach may need to be fine-tuned in terms of architecture and parameter differences in order to achieve higher results. For the future, big data and big network architectures could be applied to different body organ fracture images to provide CAD systems for them.

## Figures and Tables

**Table 1:** Recent work on automatic fracture detection using machine learning and deep learning techniques.

Fracture Type	Image Type	Feature Extraction Model	Classifier Used	Accuracy	Year
Leg bone fracture [23]	X-ray	Harris algorithm	Decision tree (DT) and KNN	82%	2018
Rib fracture [24]	CT scan	-	3D DenseNet	86.9%	2021
Skull fracture [25]	X-ray		YOLOv3	91.7%	2022
Osteoporotic vertebral fractures [26]	CT scan	ResNet34	CNN/LSTM)	89.2%	2018
Fracture detection [27]	X-ray	-	CNN	95.4%	2018
Wrist fracture [28]		Inception-ResNet version	Faster R-CNN	62.04%	2019
Lumbar vertebra compression fracture [29]	X-ray	Post-driven Learning	DL techniques and level-set methods (Pose-net and M-net)	91.60%	2020
Distal radius fractures [30]	Distal Radius Fractures	-	Random forest (RF)	91.4%	2017
Arm fracture [31]	X-ray	-	YOLOv4	81.91%	2021
Thigh fracture [32]	X-ray	-	Dilated convolutional feature pyramid network (DCFPN)	82.1%	2019
Orbital blowout fractures [33]	CT scan	Inception-V3 convolutional neural network (CNN)	Inception V3 CNN and XGBoost	92%	2019
Fracture detection [34]	X-ray	-	BPNN, SVM, and PNN	92.3%	2018
Hip fractures [35]	PXR	- Grad-CAM	Deep convolutional neural network (DCNN)	91%	2019
Bone dracture [36]	X-ray	-	Deep CNN (Adam optimizer and softmax)	92.44%	2020
Wrist dractures [37]	X-ray	-	Convolutional neural network (CNN)	96%	2018
Bone fracture [38]	X-ray	Hough transformation	KNN and SVM	90%	2020
Arm fracture [39]	X-ray	ResNet	Fast R-CNN		2020
Elbow fractures [40]	X-ray	-	CNN Xception (Vision model)	88%	2019
Wrist fraction [41]	X-ray	VGG-19 and ResNet	Deep CNN using VGG-19, ResNet	87.8%	2019
Fracture detection [42]	X-ray	-	DCNN	91.5%	2018
Wrist fracture [43]	X-ray	-	YOLOV4	96.5%	2022
Binary fracture [44]	X-ray	Binary entropy function	Convolutional neural network (CNN)	Hands 80.45% Legs 84.75% Ribs 80.65% Other 86.75%	2019
Bone fracture [45]	X-ray	2D DWT	Wavelet fuzzy phrases (WFP)	84%	2019

**Table 2:** The detailed information about the proposed dilated CNN.

Number	Layer Name	Activations	Kernel Size	Stride	Parameters	Feature Maps
1	Input layer	512 × 256 × 3	/	/	/	/
2	Convolutional layer (C1)	512 × 256 × 16	5 × 5	1	Weights = 5 × 5 × 3 × 16 Bias = 1 × 1 × 16	16
3	Batch normalization (B1)	512 × 256 × 16	/	/	Offset = 1 × 1 × 16 Scale = 1 × 1 × 16	16
4	ReLU (R1)	512 × 256 × 16	/	/	/	/
5	Max-pooling layer (MP1)	256 × 128 × 16	2 × 2	2	/	/
6	Convolutional layer (C2)	256 × 128 × 32	5 × 5	1 Dilation factor = 2	Weights = 5 × 5 × 16 × 32 Bias = 1 × 1 × 32	32
7	Batch normalization (B2)	256 × 128 × 32	/	/	Offset = 1 × 1 × 32 Scale = 1 × 1 × 32	32
8	ReLU (R2)	256 × 128 × 32	/	/	/	/
9	Max-pooling layer (MP2)	128 × 64 × 32	2 × 2	2	/	/
10	Convolutional layer (C3)	128 × 64 × 64	5 × 5	1 Dilation factor = 4	Weights = 5 × 5 × 32 × 64 Bias = 1 × 1 × 64	64
11	Batch normalization (B3)	128 × 64 × 64	/	/	Offset = 1 × 1 × 64 Scale = 1 × 1 × 64	64
12	ReLU (R3)	128 × 64 × 64	/	/	/	/
13	Max-pooling layer (MP3)	64 × 32 × 64	2× 2	2	/	/
14	Convolutional layer (C4)	64 × 32 × 128	5 × 5	1 Dilation factor = 8	Weights = 5 × 5 × 64 × 128 Bias = 1 × 1 × 128	128
15	Batch normalization (B4)	64 × 32 × 128	/	/	Offset = 1 × 1 × 128 Scale = 1 × 1 × 128	128
16	ReLU (R4)	64 × 32 × 128	/	/	/	/
17	Max-pooling layer (MP4)	32 × 16 × 128	2 × 2	2	/	/
18	Convolutional layer (C5)	32 × 16 × 128	5 × 5	1 Dilation factor = 16	Weights = 5 × 5 × 128 × 128 Bias = 1 × 1 × 128	128
19	Batch normalization (B5)	32 × 16 × 128	/	/	Offset = 1 × 1 × 128 Scale = 1 × 1 × 128	128
20	ReLU (R5)	32 × 16 × 128	/	/	/	/
21	Max-pooling layer (MP5)	16 × 8 × 128	2 × 2	2	/	/
22	Convolutional layer (C6)	16 × 8 × 256	5 × 5	1 Dilation factor = 32	Weights = 5 × 5 × 128 × 256 Bias = 1 × 1 × 256	256
23	Batch normalization (B6)	16 × 8 × 256	/	/	Offset = 1 × 1 × 256 Scale = 1 × 1 × 256	256
24	ReLU (R6)	16 × 8 × 256	/	/	/	/
25	Max-pooling layer (MP6)	8 × 4 × 256	2 × 2	2	/	/
26	Fully connected layer (FC)	1 × 1 × 2	/	/	Weights = 2 × 8192 Bias = 2 × 1	2
27	Softmax	1 × 1 × 2	/	/	/	/
28	Classification layer	/	/	/	/	/

**Table 3:** The detailed information about the proposed bidirectional LSTM.

Number	Layer Name	Activations	Parameters
1	Sequence input layer	2	/
2	BiLSTM	2000	Input weights = 8000 × 1 Recurrent weights = 8000 × 2000 Bias = 8000 × 2
3	Dropout layer	2000	/
4	BiLSTM	1000	Input weights = 4000 × 2000 Recurrent weights = 4000 × 1000 Bias = 4000 × 1
5	Dropout layer	1000	/
6	Fully connected layer	2	Input weights = 2 × 1000 Bias = 2 × 1
7	Softmax	2	/

**Table 4:** Dataset before and after pre-processing and augmentation.

Variables	Actual Data	Pre-Processed Data	Augmented Data
**Wrist X-ray images**	192	192	192
**Fractured images**	111	111	555
**Normal images**	82	82	410
**Dimension**	Different sizes, such as 344 × 596, 252 × 650, 328 × 624, and 264 × 622	512 × 256	512 × 256
**Format**	JPG file	JPG file	JPG file
**Total images**	192	192	965

**Table 5:** Non-Augmented Data before and after LSTM.

Methods	Accuracy	Precision	Sensitivity	Specificity	F1-Score	Kappa
**DCNN 1**	84.48%	87.50%	84.85%	84%	86.15%	68.52%
**DCNN-LSTM 1**	86.21%	87.88%	87.88%	84%	87.88%	71.88%

**Table 6:** Experiment 2 results of augmented data before and after using the LSTM.

Methods	Accuracy	Precision	Sensitivity	Specificity	F1-Score	Kappa
**DCNN-2**	86.54%	86.93%	92.17%	81.30%	89.47%	74.25%
**DCNN-LSTM 2**	88.24%	87.93%	92.17%	82.93%	90%	75.7%

## Data Availability

Not applicable.
