# Correction: Rashid et al. A Minority Class Balanced Approach Using the DCNN-LSTM Method to Detect Human Wrist Fracture. *Life* 2023, *13*, 133

**DOI:** 10.3390/life15030441

**Published:** 2025-03-12

**Authors:** Tooba Rashid, Muhammad Sultan Zia, Najam-ur-Rehman, Talha Meraj, Hafiz Tayyab Rauf, Seifedine Kadry

**Affiliations:** 1 Department of Computer Science, The University of Lahore, Chenab Campus, Gujrat 50700, Pakistan; 2 Department of Computer Science, The University of Chenab, Gujrat 50700, Pakistan; 3 Department of Human Resource Section, University of Gujrat, Gujrat 50700, Pakistan; 4 Department of Computer Science, COMSATS University Islamabad-Wah Campus, Wah Cantt 47040, Pakistan; 5 Independent Researcher, Bradford BD8 0HS, UK; 6 Department of Applied Data Science, Noroff University College, 4612 Kristiansand, Norway; 7 Artificial Intelligence Research Center (AIRC), Ajman University, Ajman 346, United Arab Emirates; 8 Department of Electrical and Computer Engineering, Lebanese American University, Byblos P.O. Box 13-5053, Lebanon

## Corrected Affiliation

In the published publication [1], there was an error regarding the affiliation for Hafiz Tayyab Rauf. The updated affiliation should be: Independent Researcher, Bradford BD8 0HS, UK. The authors state that the scientific conclusions are unaffected. This correction was approved by the Academic Editor. The original publication has also been updated.
